# Fabrication of fibrillosomes from droplets stabilized by protein nanofibrils at all-aqueous interfaces

**DOI:** 10.1038/ncomms12934

**Published:** 2016-10-11

**Authors:** Yang Song, Ulyana Shimanovich, Thomas C. T. Michaels, Qingming Ma, Jingmei Li, Tuomas P. J. Knowles, Ho Cheung Shum

**Affiliations:** 1 Department of Mechanical Engineering, the University of Hong Kong, Pokfulam Road, Hong Kong; 2 Institute for Research and Innovation (HKU-SIRI), Shenzhen 518000, China; 3 Department of Chemistry, University of Cambridge, Lensfield Road, Cambridge CB2 1EW, UK; 4 Department of Materials and Interfaces, Weizmann Institute of Science, Rehovot 76100, Israel; 5 Paulson School of Engineering and Applied Science, Harvard University, Cambridge, Massachusetts 02138, USA

## Abstract

All-aqueous emulsions exploit spontaneous liquid–liquid separation and due to their water-based nature are particular advantageous for the biocompatible storage and processing of biomacromolecules. However, the ultralow interfacial tensions characteristic of all-aqueous interfaces represent an inherent limitation to the use of thermally adsorbed particles to achieve emulsion stability. Here, we use protein nanofibrils to generate colloidosome-like two-dimensional crosslinked networks of nanostructures templated by all-aqueous emulsions, which we term fibrillosomes. We show that this approach not only allows us to operate below the thermal limit at ultra-low surface tensions but also yields structures that are stable even in the complete absence of an interface. Moreover, we show that the growth and multilayer deposition of fibrils allows us to control the thickness of the capsule shells. These results open up the possibility of stabilizing aqueous two-phase systems using natural proteins, and creating self-standing protein capsules without the requirement for three-phase emulsions or water/oil interfaces.

## Abstract (web summary)

All-aqueous emulsions are useful for delivering and processing biomolecules, but their stability is constrained by low interfacial adsorption energy. Song *et al*. solve this problem using protein nanofibrils that form a crosslinked network, whose stability is superior to conventional colloidal capsules.

## Graphical abstract

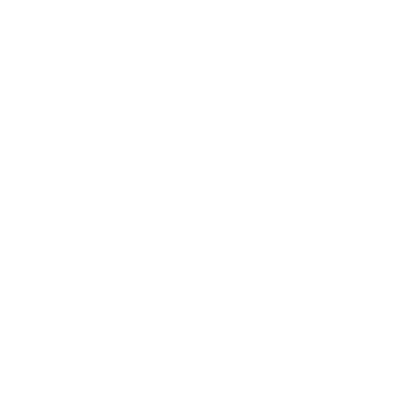

Synthetic capsules, such as polymersomes^1^,^2^ and colloidosomes^3^,^4^ have exhibited great promise in the development of artificial cells^4^, the synthesis of functional biomaterials^5^ and the manufacturing of pharmaceutical products^6^. Interfaces provided by emulsion droplets^1^,^3^, air bubbles^7^ and solid particles^8^ have been introduced as templates for the formation of capsules with designed structures^9^, tunable permeability^10^ and smart functionalities^11^. However, the stability and activity of biomolecules, including lipids and proteins can be compromised by lipid oxidation^12^ or protein denaturation^13^ at the interfaces of non-aqueous phases commonly used in encapsulation technologies, a key factor stimulating investigations of all-aqueous emulsions where both the dispersed and the continuous phases are water-based and thus biocompatible.

Water-in-water (W/W) emulsions consist of droplets formed by the phase separation of two immiscible aqueous phases^14^. In biology, such emulsions can be generated through the phase separation of protein and RNA molecules in an aqueous environment; examples include the membrane-less organelles in the cytoplasm of germ granules^15^ and nucleoli^16^. Compared with oil-containing emulsions, W/W emulsions are particularly biocompatible for the storage and processing of biomolecules^17^, the delivery of bioactive agents^18^ and are increasingly used as biomimetic templates for the synthesis of hydrogel particles^19^ or as cell-mimicking matrices^20^.

Despite these numerous applications and their inherent superior biocompatibility, the ultralow interfacial tensions^21^ characterizing all-aqueous emulsions—which typically range from 10^−7^ to 10^−4^ N m^−1^—represent the primary constraint that limits the adsorption of surface-active species at W/W interfaces and hence the stabilization of W/W emulsions. In particular, nanoparticles can dynamically adsorb to and detach from W/W interfaces, leading to increased stability by lowering the free energy of the interface, but at very low surface tensions, this colloidosomal stabilizing effect is strongly diminished^22^. Larger colloidal particles, such as *β*-lactoglobulin protein particles^23^ and PEGylated phospholipid^24^, can adsorb more strongly even to interfaces with very low surface tensions, but their diameter must typically exceed ∼130–170 nm (refs 23, 24). These observations suggest that the stabilization of W/W emulsions requires a threshold adsorption energy to counteract the Brownian motion of surface-active compounds^23^. W/W interface stability can, in principle, be improved by increasing the size of the stabilizing nanoparticles^22^,^23^,^24^ or by optimizing their geometry, for example, by using nanotubes and nanoplates^25^,^26^,^27^,^28^,^29^,^30^. However, the packing of large colloids at W/W interfaces is often associated with geometrical constraints that result in low overall coverage ratios of 20–40%^22^,^23^,^24^, thus leading to challenges in fabricating regular and robust capsules^31^. We therefore hypothesize that the growth of active compounds pre-seeded at W/W interfaces would combine the benefits from the high surface affinity of large colloids and their effective packing at all-aqueous interfaces. Using amyloid nanofibrils as a model, we expect that the growth of nanofibrils and the subsequent formation of two-dimensional (2D) networks would provide a new driving force for the stabilization of W/W emulsions.

In this paper, we fabricate robust colloidosomes, which we term ‘fibrillosomes', by stimulating the formation of multilayered fibrils at all-aqueous interfaces. We show that the non-covalently crosslinked fibrillosomes stabilize all-aqueous emulsions even below the threshold interfacial tension set by thermal energy. Moreover, the covalent crosslinking of such shells allows them to be self-standing even in the total absence of an interface once they have been formed. Our findings suggest new biomimetic strategies to control the fabrication of synthetic vesicles by using naturally occurring proteins in an all-aqueous process.

## Results

### Stabilization of all-aqueous emulsions by amyloid nanofibrils

**Figure 1:**
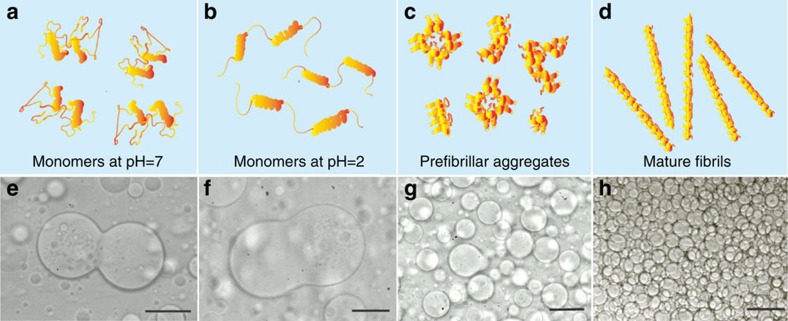
Stability of dextran-in-PEG emulsions by adding lysozyme in different aggregation stages. (**a**–**d**) Graphical representation of lysozyme protein assemblies in different stages of their fibrillization process: monomers at pH=7 (**a**), monomers at pH=2 (**b**), prefibrillar aggregates (**c**) and mature fibrils (**d**). (**e**–**h**) The corresponding optical micrographs show the different stabilization properties of lysozyme aggregates in the indicated stages of fibrillization. All incubation times corresponding to specific panels. Scale bars, 50 μm.

**Figure 2:**
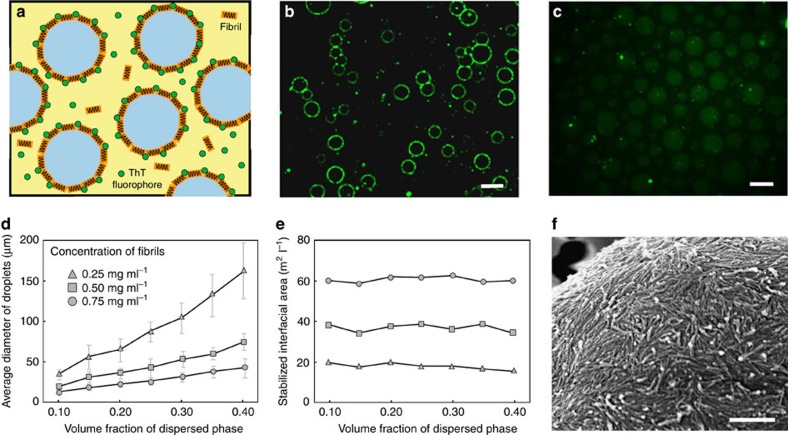
A monolayer of fibrils is efficiently adsorbed at the W/W interface. (**a**) Scheme and (**b**) fluorescence microscopy image of ThT-dyed lysozyme fibrils accumulated at the interface of W/W emulsion droplets. Scale bar, 20 μm. (**c**) In the absence of fibrils, weak ThT fluorescence is observed in the dextran-rich droplet phase. Scale bar, 20 μm. (**d**) The average diameter of stabilized W/W emulsion droplets increases linearly with the volume fraction of the emulsion phase. Error bars represent the s.d. (**e**) The total interfacial area stabilized by fibrils increases almost linearly with the fibril concentration and remains constant when changing the volume fraction of the droplet phase. (**f**) An SEM image confirms that lysozyme fibrils deposit as a monolayer at the emulsion interfaces. Scale bar, 500 nm.

Fibril formation of hen egg white lysozyme is triggered by thermal incubation at 60 °C (ref. 32). The conversion of soluble precursor proteins into mature fibrils is observed over several days; the resulting fibrils have a thickness of about 15 nm and an average length of 600 nm (see Supplementary Fig. 1). To investigate the connection between fibril formation and the stability of W/W emulsions, we prepare protein samples at different stages of their growth process by varying the time of incubation^33^,^34^, as illustrated in the scheme of Fig. 1a–d (see Methods). The W/W emulsions are generated by dissolving 2 wt% dextran and 8 wt% polyethylene glycol (PEG) into an aqueous suspension containing the different protein samples. For the same total mass concentration of proteins, we find dramatic differences in the way protein samples incubated for different times stabilize the W/W emulsions. In particular, lysozyme monomers are predominantly localized in the dextran-rich phase and only weakly enriched at the W/W interface^35^. This finding is consistent with the fact that the adsorption energy of lysozyme monomers, which is given by 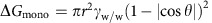 (refs 22, 23), counteracts only <1% of the thermal motion energy due to their small radius, *r*=5 nm (ref. 36), as well as the low interfacial tension *γ*_W/W_ of the W/W interface (see Supplementary Note 1). Thus, lysozyme monomers cannot form an effective physical barrier to prevent coalescence of W/W droplets, in agreement with our observation that W/W emulsions containing native or unfolded lysozyme underwent coalescence as fast as those without any protein additives (Fig. 1e,f). Similarly, early aggregated forms of lysozyme obtained after 17 h of incubation^32^ do not enhance significantly the emulsion stability (Fig. 1g). By contrast, mature lysozyme fibrils stabilize W/W emulsions in a highly robust manner. This dramatic effect is confirmed by the almost complete absence of droplet coalescence even for concentrated W/W emulsions (Fig. 1h). Remarkably, this stabilizing effect is observed at concentrations of lysozyme fibrils as low as 0.025 wt%. To understand the exceptional emulsion stability resulting from the addition of protein nanofibrils, we first examine the distribution of fibrils in the dextran-in-PEG emulsion droplets by labelling the fibrils with thioflavin T (ThT)^37^. Fluorescence microscopy reveals that the fibrils are located at the emulsion interface, while both the emulsion and the continuous phases show only negligible fluorescence signal (Fig. 2a–c and Supplementary Fig. 2).

To confirm the role of fibrils in stabilizing W/W interfaces, we varied the total interfacial area by increasing the volume fraction of the dextran-rich dispersed phase, *δ*, from 10 to 40% (see Methods), while keeping the concentration of fibrils, *ω*_*f*_, constant at 0.025 wt%, 0.05 wt%, and 0.075 wt%, respectively. Emulsion droplets with a well-defined size distribution were generated by applying a constant spinning rate and their stability was monitored as a function of time by optical microscopy. Because the generated droplets have a similar size distribution but the concentration of added fibrils remains constant, the average diameter *D* of droplets increases with increasing *δ* as a result of coalescence (Fig. 2d), thus confirming the stabilizing role of fibrils. Another important parameter is the total interfacial area *S* stabilized by fibrils, which is given by *S=6δ*/*D*. Since the total area of stabilized interface is controlled by the amount of added fibrils, the stabilized interfacial area was observed to be independent of the volume fraction of the emulsion phase, as shown in Fig. 2e.

Next, to determine the minimum thickness of the fibril layer required to stabilize W/W interfaces, we varied the total concentration ω of fibrils. We find that the stabilized surface area increased linearly with the concentration of fibrils *ω*_*f*_, (Fig. 2e); the slope of this linear relationship provides an estimate of the surface area stabilized fibrils, leading to a value of 80 m^2^ of stabilized interfacial area per gram of added fibrils (Supplementary Note 2). This area density of fibrils corresponds to an average thickness of the fibril layer of about 17 nm. Because the thickness of individual fibril is ∼15nm, our results show that fibrils adsorb at the interface to form a monolayer. The monolayer adsorption of fibrils at the W/W interface is also confirmed from the electron microscopic images in Fig. 2f. The coverage ratio of the monolayer is ∼60–70%.

### Mechanism of stabilization

**Figure 3:**
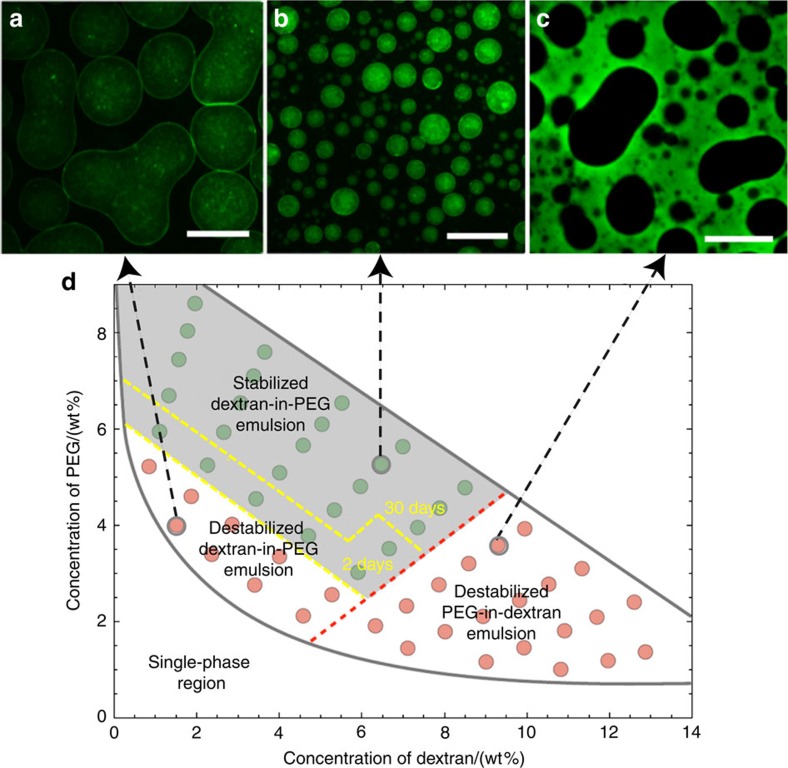
Compositions of W/W emulsions controls the stabilizing roles of fibrils. By mixing 0.05 wt% fibrils with W/W emulsions at different compositions, a phase diagram distinguishes three regions depending on emulsion stability, including destabilized dextran-in-PEG emulsions (**a**), stabilized dextran-in-PEG emulsions (**b**) and destabilized PEG-in-dextran emulsions (**c**). The representative fluorescence images of each region are shown in panels (**a**–**c**), where the dextran-rich phase is labelled in green. Scale bars, 50 μm. (**d**) The points in between the yellow dashed lines represent metastable dextran-in-PEG emulsions, which are stable for only 3–14 days.

Since the assembly of fibrils relies on the presence of W/W interfaces, fibrils should cease to stabilize W/W emulsions when the emulsion composition approaches the lowest limit for phase separation. To test this prediction, we varied the concentration of dextran and PEG in the mixture and tested emulsion stability in the presence of 0.05% fibrils. For a volume fraction of the dextran-rich phase of <55%, the formation of dextran-in-PEG emulsion droplets is kinetically favoured after vortex mixing, whereas for a volume fraction of more than 55% PEG-in-dextran emulsion is formed. We were able to distinguish the two kinds of emulsion by labelling dextran molecules with fluorescein isothiocyanate (FITC). For the dextran-in-PEG emulsions, destabilization occurred near the phase boundary separating one and two-phase regions (Fig. 3a); stabilized W/W droplets (Fig. 3b) are shown by the grey region in the phase diagram. The boundary line dividing the regions of stabilized and non-stabilized dextran-in-PEG emulsions is a tie line of the equilibrium phases of dextran and PEG, suggesting that the interfacial tension dominates this destabilization (see the yellow dashed line in Fig. 3). After a few weeks, the emulsions with compositions near the boundary line gradually broke, whereas the droplets with compositions far above the boundary line remained stable for one month.

The stability of dextran-in-PEG emulsions can be explained by the adsorption energy of fibrils at the W/W interface. By neglecting the finite thickness of the interface, the change in free energy associated with the adsorption of a single cylindrical fibril at the W/W interface can be expressed as (refs 38, 39)


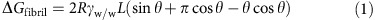


where 2*R* is the diameter of fibrils, *γ*_W/W_ is the surface tension of the W/W interface, *L* is the average length of fibrils, and *θ*=142±7° is the wetting angle of the dextran-rich phase to the fibril (Supplementary Fig. 3 and Supplementary Note 3). Using this result^38^,^39^, we conclude that a minimum interfacial tension of *γ*_*th*_*=*6 × 10^−6^ Nm^−1^ is required to stabilize W/W emulsion with a monolayer of fibrils. This threshold surface tension corresponds to an adsorption energy of 5 × 10^−21^ J, which approximately equals the thermal energy *kT*=4 × 10^−21^ J at room temperature. This result suggests that the stability of dextran-in-PEG emulsions is explained by the dynamic adsorption and desorption of fibrils at the W/W interface.

Unlike fibril-coated dextran-in-PEG emulsions, fibril-coated PEG-in-dextran emulsions were observed to coalesce above *γ*_*th*_(see Fig. 3c). Hence, the high stability of dextran-in-PEG emulsions cannot be explained only by the adsorption energy. Indeed, the large wetting angle *θ* implies that most of the fibril surface is in contact within the PEG-rich phase so that the desorption energy for fibrils to enter the dextran-rich phase is about 25 times higher than that for partitioning into the PEG-rich phase (see Supplementary Fig. 4 and Supplementary Note 4). This ‘disaffinity' of fibrils to the dextran-rich phase effectively provides a physical barrier that prevents the direct contact between adjacent dextran-in-PEG droplets, explaining the enhanced stability of dextran-in-PEG emulsions relative to PEG-in-dextran emulsions.

### Enhanced emulsion stability by 2D multilayer fibril networks

**Figure 4:**
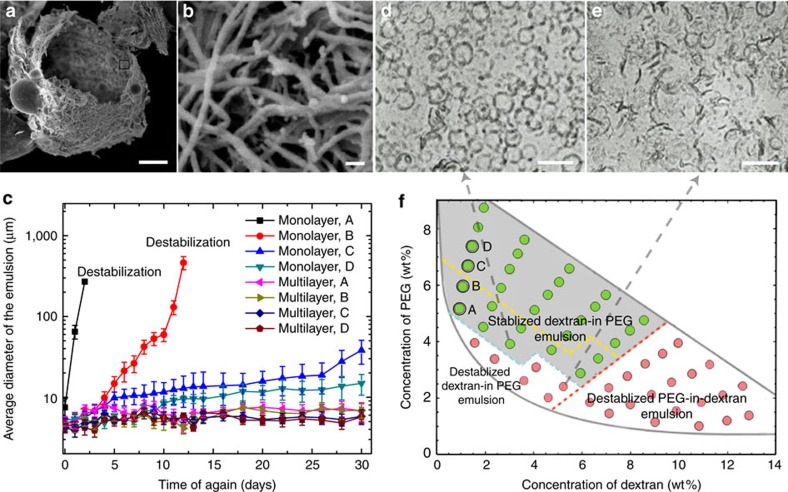
Growth of fibril layers at the W/W interface enhances the stability of W/W emulsions. (**a**,**b**) SEM images of lysozyme capsules composed of multilayers of fibrils. W/W emulsions coated with fibril monolayers are used as the seeding templates, and the formation of multilayer fibrils is induced by the fibrillization of monomeric lysozyme. Scale bars; 2 μm (**a**); and 50 nm (**b**). (**c**) A comparative study of the size of emulsion droplets stabilized by a monolayer and a multilayer of fibrils as a function of aging time. Error bars represent the deviations.d. of the droplet diameters. The different compositions of the tested emulsions (A–D) are shown in **f**. (**d**,**e**) Representative microscopic images of the multilayer fibril stabilized and destabilized dextran-in-PEG emulsions are shown. Scale bars, 20 μm. (**f**) Compared with fibril monolayers, fibril multilayers enhance the stability of W/W emulsions over 30 days, as suggested by the enlarged stability region in the phase diagram (grey area) corresponding to emulsions stabilized by the fibril multilayer. Emulsions stabilized by a fibril monolayer remained stable for 30 days only in the area above the yellow dashed lines.

The stabilization of W/W emulsions at ultra-low interfacial tensions, *γ<γ*_*th*_, is compromised by the lowered surface affinity of fibrils; however, the formation of 2D multiplayers of fibrils localized at the W/W interface provides a new solution to further enhance emulsion stability. In this approach, a monolayer of nanofibrils (0.05 wt%) covering the stable dextran-in-PEG emulsions are used to seed further fibril deposition at the W/W interface. To facilitate the adsorption of fibrils, we add 0.5 wt% lysozyme monomers to the emulsion mixture and maintain favourable conditions (the mixture is incubated at 60 °C, pH=2) for the conversion of monomeric lysozyme into mature fibrils. As the fibrils near the W/W interface grew, the thickness of the fibril layers increases, as confirmed by the scanning electron microscopy (SEM) images in Fig. 4a. The total amount of fibrils adsorbed at the droplet interface can be controlled by varying the concentration of the added monomer solution from 0.25 to 2 wt% (see Supplementary Fig. 5). After 36 h, we diluted the stable dextran-in-PEG emulsions until the interfacial tension fell below *γ*_*th*_. The stability of the diluted emulsion droplets is established by following variations in the droplet size as a function of time for over one month. Compared with dextran-in-PEG emulsions coated with fibril monolayer, multilayered fibrils better stabilize the emulsion droplets, as demonstrated by the overall small change of the average droplet diameter (Fig. 4c and Supplementary Fig. 6). Accordingly, the region of the phase space representing the dextran-in-PEG emulsions that are stable for over 30 days expands (Fig. 4d-f). Remarkably, multilayered fibrils can stabilize some dextran-in-PEG emulsions with an interfacial tension below *γ*_*th*_, as a result of the formation of 2D fibril networks. This result suggests that the formation of nanofibril multilayers overcomes the fundamental limitation of the threshold interfacial tension *γ*_*th*_ as defined by the thermal energy.

### Robust fibrillosomes templated by all-aqueous interfaces

**Figure 5:**
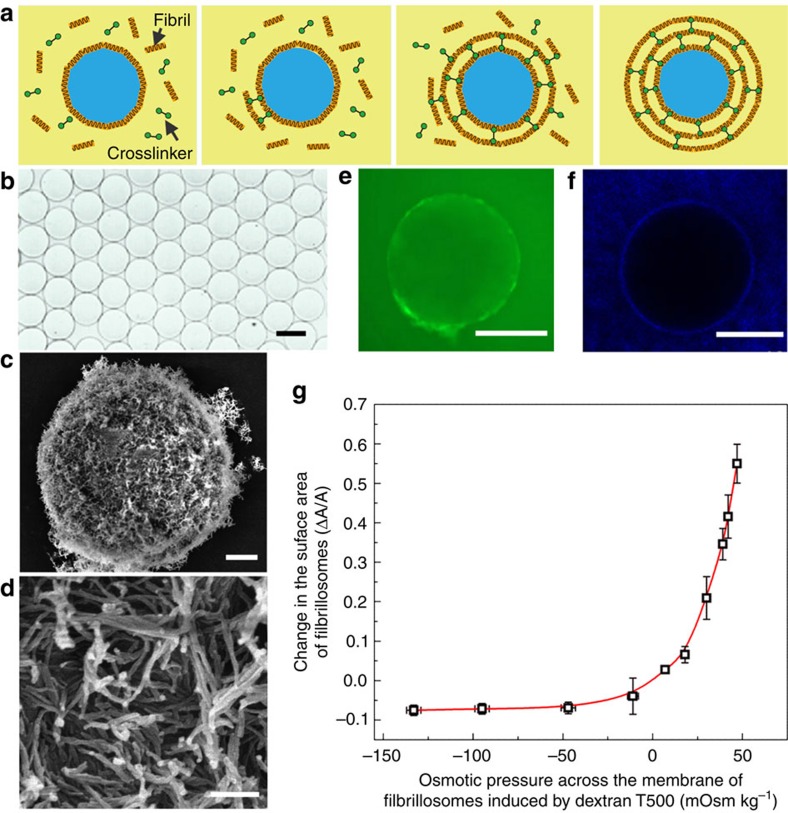
Protein fibrillosomes templated from W/W emulsions. (**a**) Schematics of the formation of protein fibrillosomes by crosslinking fibril-coated droplets. (**b**) Optical microscope images of monodisperse fibrillosomes obtained after replacing the continuous phase with the same liquid inside the fibrillosomes. Scale bar, 100 μm. (**c**,**d**) SEM images of fibrillosomes with their walls consisting of amyloid fibrils. Scale bars; 2 μm (**c**); and 200 nm (**d**). (**e**) FITC-dextran macromolecules with hydrodynamic diameters of around 30 nm can penetrate through the membrane of fibrillosomes. Scale bar, 200 μm. (**f**) Fluorescent nanoparticles with diameters of 50 nm fail to penetrate the fibrillosomes. Scale bar, 200 μm. (**g**) The fibrillosomes exhibit excellent elasticity and robustness upon osmotic swelling and shrinking. The concentration of dextran in the continuous phase was varied from 4 to 25 wt%, while the dextran concentration inside the fibrillosomes was kept at 15 wt%, generating an osmotic pressure across the membrane. Error bars represent the s.d.

Finally, we investigate the use of fibril-coated emulsions as templates for the construction of covalently linked protein colloidosomes (Fig. 5a). To this end, we first adopt an electrospray approach to generate monodisperse W/W droplets with a diameter of around 100 μm (Fig. 5b). Next, lysozyme fibrils templated at the interface of the dextran-in-PEG emulsions are covalently crosslinked with the non-adsorbed fibrils by using glutaraldehyde, resulting in the formation of 2D fibril networks (Fig. 5c,d). Compared with non-covalently crosslinked capsules (Fig. 4), the covalently crosslinked fibrillosomes are mechanically robust and remain stable after the PEG-rich continuous phase is replaced with the same solution inside the droplet (Fig. 5b). The semi-permeability of the fibrillosomes is characterized by separately adding fluorescent FITC-dextran and nanoparticles from the continuous phase and testing their penetration into the fibrillosomes. While macromolecules of FITC-dextran (*M*_w_=500,000) with a hydrodynamic diameter of 30 nm can penetrate slowly the fibrillosomes (Fig. 5e), larger fluorescent nanoparticles with diameter of 50 nm fail to penetrate the membrane (Fig. 5f). When rapidly transferred into a hypertonic 25% dextran solution (181 mOsm kg^−1^), the fibrillosomes encapsulating 15% dextran (48 mOsm kg^−1^) shrink and buckle (Supplementary Fig. 7). When transferred into a hypotonic 4% dextran solution (1 mOsm kg^−1^), the membrane area of the fibrillosomes increases by 60% without rupture (Fig. 5g). These properties enable the use of our robust fibrillosomes in the near physiological environment and in cell-culture media, such as the PBS solution.

## Discussion

In this paper, we present an approach for using W/W emulsion droplets as templates for the assembly of monolayer and multilayer protein nanofibrils. This approach allows us to stabilize all-aqueous emulsions even with ultra-low interface tensions. The stabilization originates from the combination of the strong interface affinity and the high aspect ratio of the protein fibrils that results in a higher surface coverage compared with spherical colloids. Moreover, using the seeded growth of fibrils near the W/W interface, we demonstrate the fabrication of multilayered fibrillosomes, 2D nanofibril networks arising from intra-colloidal interactions. Robust fibrillosomes are further prepared by covalently crosslinking the multilayered fibrils, yielding stretchable capsules with semi-permeability. These results open up the possibility of fabricating highly robust vesicles by using the mild templates of all-aqueous interfaces. Compared with capsules formed by using the templating action of water-in-oil-in-water double emulsions, our approach avoids the use of organic solvents, which can denature proteins^12^. This approach, therefore, has the potential of facilitating the fabrication and use of protein-based materials in a biocompatible manner, for instance, for the encapsulation and delivery of bioactive species, such as antibodies or enzymes^40^.

## Methods

### Synthesis and characterization of protein fibrils

A 20 mg/ml solution of hen egg white lysozyme (Sigma Aldrich) was prepared by dissolving 0.2 g of protein into 10 ml of solution containing 2 ml of 1 M HCl, 6 ml of 10 mM HCl and 2 ml of 10 mM NaCl (with the pH pre-adjusted to 2 using HCl). The solution was filtered (mesh size 0.2 μm) to yield a solution of monomeric lysozyme. To induce fibril formation, the solution was incubated at 57–65 °C and stirred at 550 r.p.m. for 60–70 h (ref. 34). The resulting fibril solution was diluted to 0.025–0.2 wt% before use. The height of lysozyme fibrils was measured by Atomic Force Microscopy (AFM, Bruker Multimode 8). To this effect, a 0.01 wt% fibril suspension was drop-cast onto a mica substrate and dried at room temperature. AFM imaging was conducted at a scan rate of 0.3 Hz in tapping mode. The length of lysozyme fibrils was characterized by scanning electron microscopy (SEM, Hitachi S-4800). The average length of fibrils was measured by counting the pixels of at least 50 fibrils from at least 10 images. The prefibrillar oligomers of lysozyme were prepared by incubating the monomer solution for 17 h without stirring.

### Characterization of emulsion stability

A total of 5–15 wt% dextran (*M*_w_=500 kDa) and 4–9 wt% PEG (*M*_w_=20 kDa) were dissolved into a 0.025–0.08 wt% fibril suspension. The volume fraction of the dextran-rich emulsion phase was varied from 10 to 40% by changing the emulsion compositions along the tie line connecting the equilibrium phases of dextran and PEG in the phase diagram, as denoted by the dots in Fig. 3d. In order to form emulsion droplets with uniform diameters, the spinning rate of homogenization was kept at 400–600 r.p.m. for 2 min. Emulsion stability was monitored daily until the demixing of the two bulk phases was observable under a fluorescence microscope. Fibrils were labelled with 10-20 μM Thioflavine T (ThT) and incubated in the mixture solution at 60 °C for 15 min. The fibrils adsorbed at the emulsion interface were imaged under a fluorescence microscope (ECLIPSE TE 2000-U). A blue laser with a maximum excitation wavelength of 445 nm was used to excite and detect the ThT-labelled fibrils. An optical filter was used for imaging the fluorescence emitted from the ThT-labelled fibrils to block noise signals with wavelength below 480 nm.

### Measurement of W/W interfacial tension

The interfacial tension between two immiscible aqueous phases was measured using a spinning drop tensiometer (Kruss Site 100). The dextran-rich phase, with a higher density than the PEG-rich phase, was used as the continuous phase for spinning. PEG-rich droplets were injected into a glass tube filled with the dextran-rich phase and stretched into ellipsoid shape upon rotating at 3,000–6,000 r.p.m. When the length of the ellipsoid droplet was four to six times longer than the width, the rotational speed and the width of the droplets were recorded to calculate the interfacial tension^21^.

### Seeded growth of fibrils at the droplet interface

A total of 0.25–2 wt**%** lysozyme monomers were dissolved in a mixture solution containing 2 ml of 1 M HCl, 6 ml of 10 mM HCl and 2 ml of 10 mM NaCl as a precursor solution. Afterwards, we added 0.1% fibrils, 3% dextran and 8% PEG into the precursor solution and mixed at 500 r.p.m. for 1 min. This enabled the formation of stable dextran-in-PEG emulsions coated with a monolayer of fibrils. Finally, we incubated the emulsion mixture with monomers at 60 °C for 72 h. To quantify the amount of fibrils converted from monomeric protein, fibrils were stained with ThT and their fluorescence intensity is measured using a plate reader.

### Fabrication of protein fibrillosomes

To form fibrillosomes with uniform diameters, we employed an electrospray approach to generate W/W emulsion droplets^19^. An emulsion phase containing 10 wt% dextran and 0.1 wt% fibrils was electrosprayed in air to form droplets. The applied voltage was 2.6 kV and the distance between the anodic nozzle and cathode was 1.5 mm. The droplets were collected in a petri dish containing a PEG-rich continuous phase (8 wt% PEG, 0.15 wt% fibrils and 2.5 wt% glutaraldehyde). After reaction at 65 °C for 12 h, robust fibrillosomes were formed.

### Characterization

We slowly injected a 15% dextran solution into the emulsion mixture, and extracted the PEG-rich continuous phase from the top, so that the fibrillosomes were immersed in the dextran-rich phase. To test the permeability of the fibrillosomes, 0.1 wt% FITC-dextran (Mw=500,000, Sigma) and 0.1 wt% amine modified latex beads (50 nm in diameter, Sigma) were added from the continuous phase and mixed with fibrillosomes. After 24h of incubation, images were taken by fluorescence microscopy. To test the swelling and shrinking properties of fibrillosomes, we transferred the fibrillosomes into dextran solutions with concentrations ranging from 4 to 25 wt%. The osmolarity of the external dextran solution was measured by osmometer (Model 3320, Advanced Instrument, Inc.). After osmotic swelling or shrinking, the diameter of fibrillosomes was measured from bright field microscope images. The surface morphology of protein fibrillosomes was characterized under SEM. To preserve the original morphology, fibrillosomes were first dehydrated by 95% ethanol and then dried in the liquid carbon dioxide at its super critical point. The immobilized protein fibrillosomes were coated with a thin film of gold and observed under SEM (Hitachi S4800 FEG, 5 kV)

### Data availability

The authors declare that the data supporting the findings of this study are available within the article and its Supplementary Information files.

## Additional information

**How to cite this article:** Song, Y. *et al*. Fabrication of fibrillosomes from droplets stabilized by protein nanofibrils at all-aqueous interfaces. *Nat. Commun.* **7,** 12934 doi: 10.1038/ncomms12934 (2016).

## Supplementary Material

Supplementary Information Supplementary Figures 1-7 and Supplementary Notes 1-4
