# Supplementary material for: Fabrication of fibrillosomes from droplets stabilized by protein nanofibrils at all-aqueous interfaces
Source: Nat Commun. 2016 Oct 11;7:12934. doi: 10.1038/ncomms12934 (PMC5062572; doi:10.1038/ncomms12934)
Supplement: Supplementary Information — Supplementary Figures 1-7 and Supplementary Notes 1-4 [file ncomms12934-s1.pdf]

Supplementary information

Supplementary Figure 1

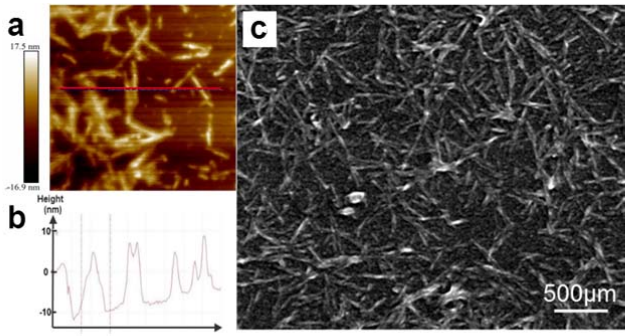

Supplementary Figure 1 l Characterization of the lysozyme fibrils by atomic force microscopy (AFM) and scanning electron microscopy (SEM). (**a**) **&** (**b**) The height of lysozyme mature fibrils is 15 nm on average, measured by AFM. (**c**) A SEM image reveals that the average length of lysozyme fibrils is around 600 nm.

Supplementary Figure 2

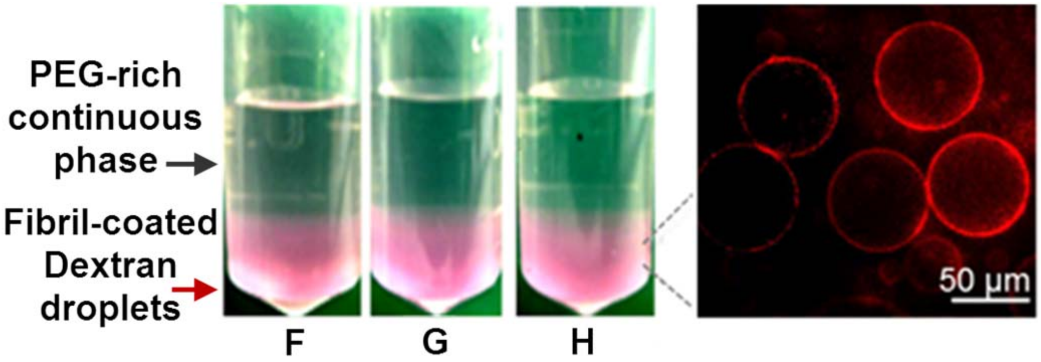

Supplementary Figure 2 | Supporting evidences showing that almost complete accumulation of

0.05 wt% lysozyme fibrils at the interface of W/W emulsion droplets. (a) In a solution mixture containing Nile Red-labelled fibrils and dextran-in-PEG emulsion droplets, a colorless PEG-rich continuous phase is observed after the sedimentation of dextran-in-PEG emulsion droplets. The concentrations of dextran in the dextran-rich bottom phases are 13.9%, 14.3% 15.0% for samples F, G and H, respectively. **b,** A fluorescence microscope image confirms that dyed fibrils accumulate at the droplet interface.

When the amount of fibrils is insufficient to cover the created interfacial area of W/W emulsions, almost all the fibrils go to the interface and only a very little amount of fibrils go to the bulk phases. Experimentally, fibrils with a low concentration of less than 0.08 wt% is labelled with Nile red for direct imaging of the distribution of fibrils under natural light. After homogenization, stable dextran-in-PEG droplets sink down to the bottom of the vial due to gravity. Consequently, a colorless PEG-rich phase appears on the top (Supplementary Figure 2). When a blue laser is used to excite Nile Red, a red color is emitted from the W/W interface, suggesting the accumulation of fibrils at the interface of the emulsion droplets.

Supplementary Figure 3

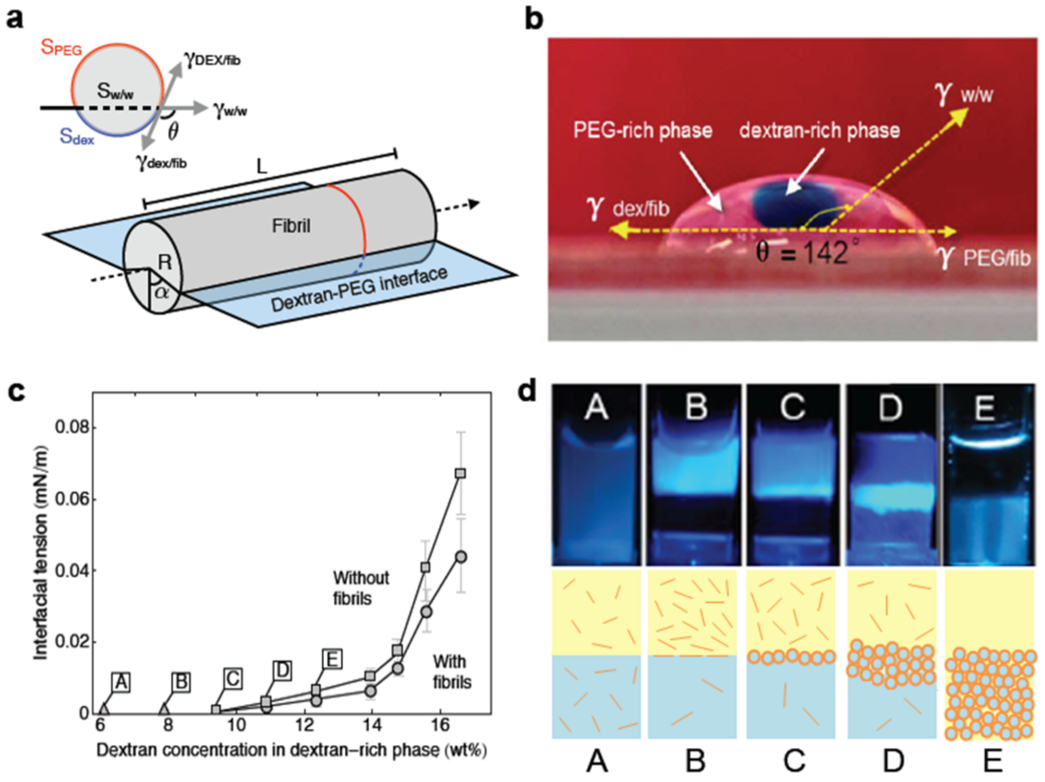

Supplementary Figure 3 | Effect of interfacial tension on the stability of the fibril-coated dextran-in-PEG emulsions. (**a**), Graphical representation of a single fibril adsorbed at the W/W interface. (**b**), The wetting angle of a dextran-rich droplet at the interface between a fibril film and a PEG-rich phase is about 142°. (**c**), Interfacial tension between two aqueous phases, $\gamma_{W/W}$, increases as the equilibrium concentrations of the PEG-rich and dextran-rich phases increase (see square dots in figure S5c). After the adsorption of fibrils, the interfacial tension slightly reduces (round dots in figure S5c. (**d**), With decreasing interfacial tensions from sample E to A, the sedimentary dextran-in-PEG emulsions become less stable. Correspondingly, more fibrils detach from the droplet interface and migrate to the PEG-rich top phase, as indicated from the fluorescence of ThT-bounded fibrils from sample E to B. For sample E, the dextran-rich emulsions sink down at the bottom of the vial due to gravity, so the fluorescence signals are only observed at the bottom of the vial. For sample A, fibrils distribute evenly in the mixture since their

interaction with the W/W interface is weak compared to the thermal energy of a few kT.

As the interfacial tension $\gamma_{W/W}$ decreases, the desorption energy of fibrils from the W/W interface decreases, resulting in the declining stability of dextran-in-PEG emulsions. In our experiment, we reduced the interface tension by decreasing the concentration of dextran and PEG in the emulsion mixture while keeping an equal volume of the dextran-rich phase and PEG-rich phases. The dextran-in-PEG emulsions are found to destabilize faster as the interfacial tension decreases; correspondingly, more desorbed fibrils leave the droplet interface and enter the PEG-rich phase, as shown by the fluorescence of ThT-labeled fibrils from sample E to A in figure S5d. For each sample, a schematic figure is drawn below to illustrate that the decreased emulsion stability is due to the decreased adsorption of fibrils at the droplet interface.

Supplementary Figure 4

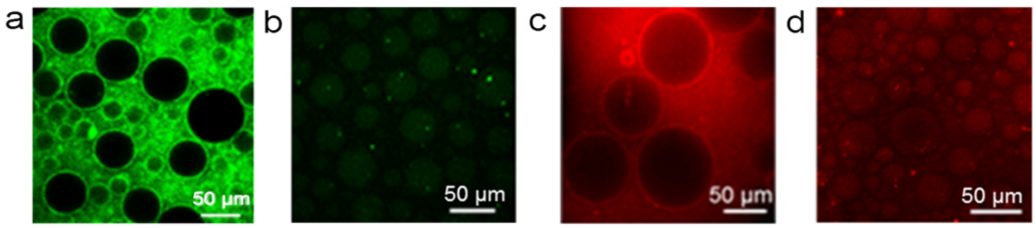

Supplementary Figure 4 | Desorbed lysozyme fibrils preferentially enter the PEG-rich phase rather than the dextran-rich phase. When the amount of fibrils added is larger than that required to cover the droplet interface, the remaining non-adsorbed fibrils preferentially enter the PEG-rich phase. To visualize the lysozyme fibrils in this condition, we labelled the fibrils with (**a**) ThT and (**c**) Nile red. Their partitioning to both the emulsion interfaces and the PEG-rich phase can be confirmed by the fluorescence microscope images. In comparison, without addition of fibrils, the dye of (**b**) ThT and (**d**) Nile red do not exhibit observable partitioning effects in either

the PEG-rich phase or the W/W interface.

Supplementary Figure 5

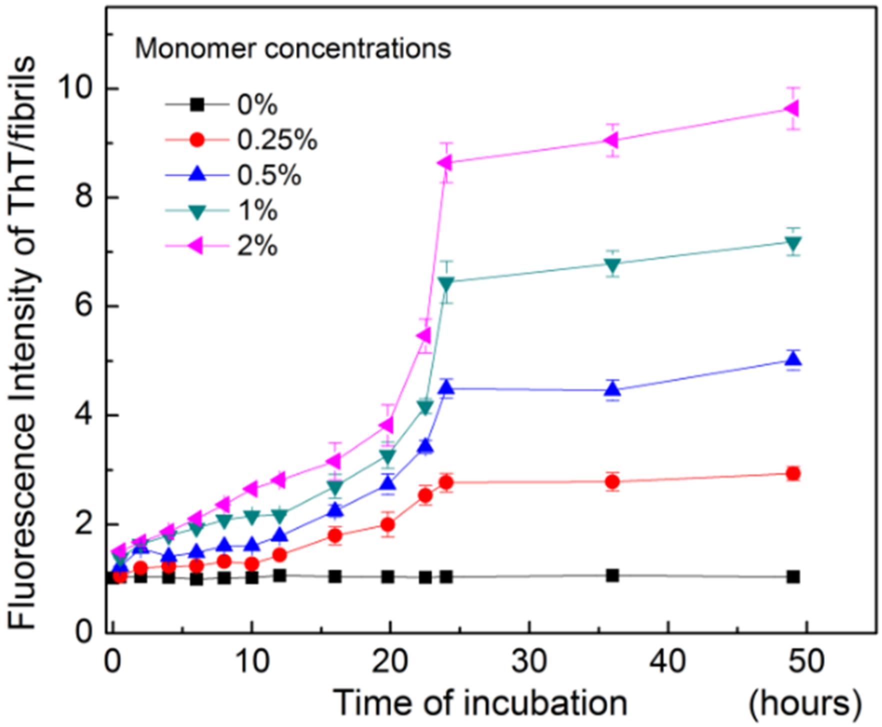

Supplementary Figure 5 | Seeded growth of the fibril layers at all-aqueous interfaces is quantitively studied by adding monomeric lysozyme at different concentrations. The total amount of fibrils at different time points is determined by measuring the intensity of fluorescence of ThT-labeled fibrils.

In our experiment, we first dissolve 2% dextran, 8% PEG and 0.05% fibrils in 200 mM HCl solution by vortex mixing, obtaining dextran-in-PEG emulsion stabilized by fibril monolayers. We then add lysozyme monomers (0 wt%- 2 wt%) into the emulsion mixture at different concentrations and incubate the mixture at 65 °C.

Supplementary Figure 6

Multilayered protein fibrils better stabilize w/w emulsions than monolayered fibrils after 30 days of aging.

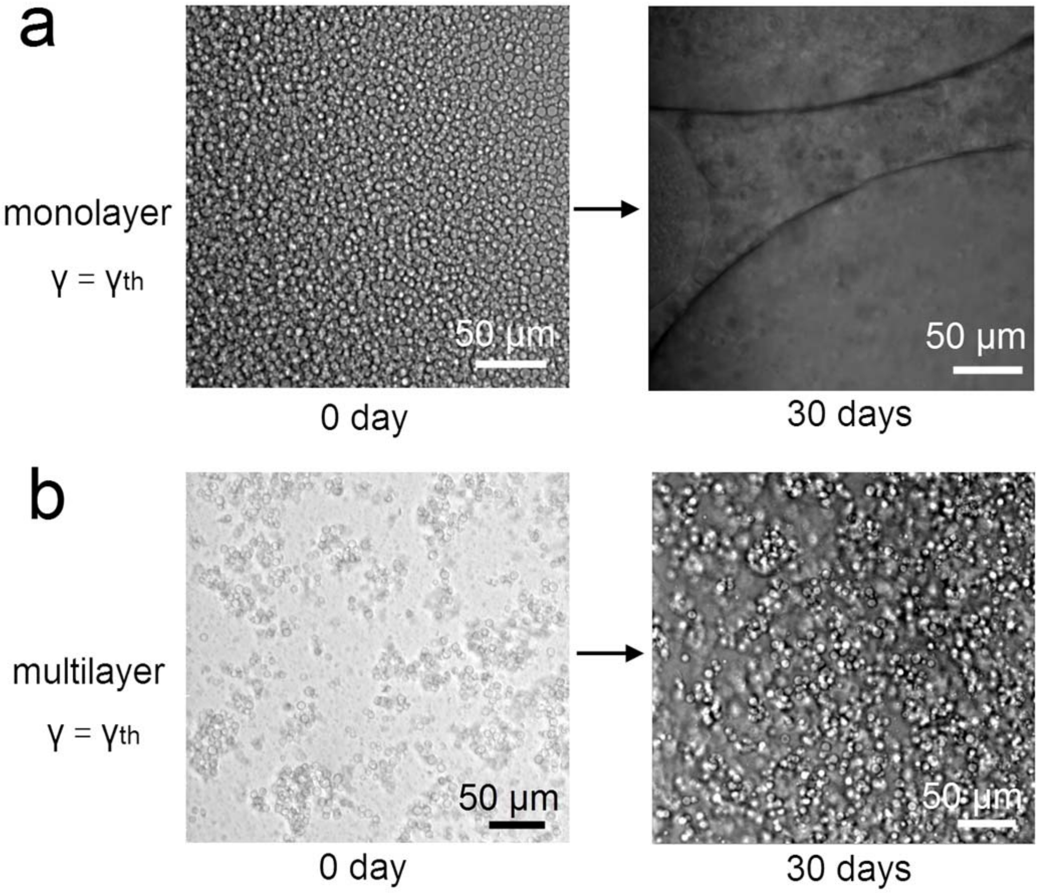

Supplementary Figure 6 | Optical microscope images of the w/w emulsions stabilized by monolayered and multilayered fibrils. (**a**) While a monolayer of fibril fails to stabilize w/w emulsion after 30 days, (**b**) the multilayered fibrils can stabilize the w/w droplets and maintain the initial droplet sizes. The interfacial tension of the w/w emulsion is kept at the threshold values for interfacial adsorption of single fibril, $\gamma=\gamma_{th}$. The compositions of the emulsion droplets are denoted by point B in the phase diagram of figure 4f in the main text.

Supplementary Figure 7

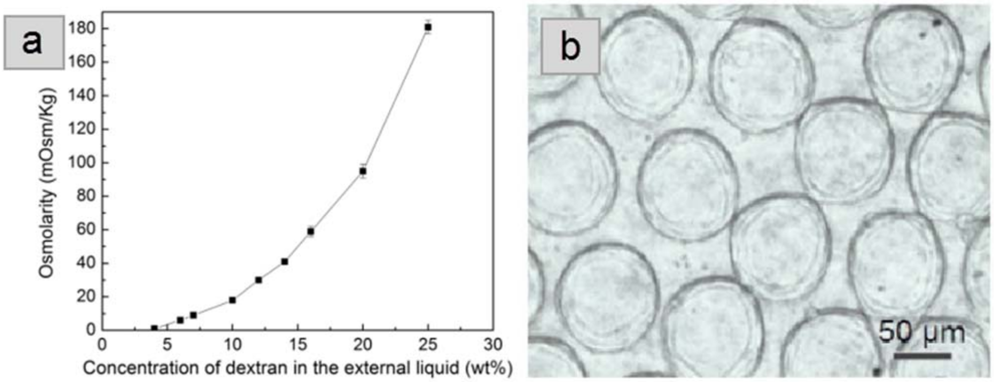

Supplementary Figure 7 | Osmotic shrinkage of the fibrillosomes (**a**) Osmolarity of the dextran solution as a function of the concentration of dextran. (**b**) An optical microscope images showing the buckling morphology of the shrinking fibrillosomes in a hypertonic dextran solution (25 wt%, 181 mOsm/Kg).

A 15 wt% dextran (48mOsm/Kg) solution is first encapsulated inside the fibrillosomes by using dextran-in-PEG emulsions as templates. To generate an osmotic pressure across the membrane of fibrillosomes, we transferred the fibrillosomes into different dextran solutions with their concentrations ranging from 4 wt% to 25 wt% (Reaching the solubility limit of dextran in water). The corresponding osmolarity of the dextran external liquid changes from near zero to 181mOsm/Kg, as shown in the supplementary figure 7a. Due to the gradient in the concentration of dextran across the wall of the fibrillosomes, an osmotic pressure appears at the very beginning. The initial osmotic pressure can be calculated by using the osmolarity of the internal dextran solution minus that of the external dextran solution. With a positive osmotic pressure, water is sucked out of the fibrillosomes, leading to the shrinkage of fibrillosomes. On the contrary, the

fibrillosomes swell in a hypotonic dextran solution. Although dextran can slowly penetrate through the membrane of fibrillosomes, water diffuses across the membrane at a much faster speed, due to their much smaller sizes. Therefore, the osmotic effect still controls the swelling or shrinkage of fibrillosomes.

Supplementary Note 1

A calculation on the adsorption energy of lysozyme monomers to the w/w interface

We treat lysozyme monomers as sphere-shaped colloidal particles with the hydrodynamic diameter of 5 nm[38]. Ignoring the curvature of the W/W interface, the created area of W/W interface can be expressed as $S_{w/w} = \pi r^2 \sin^2\theta$, and the removed dextran/fibril contact area $S_{dex} = 2\pi r^2(1-\cos\theta)$. The adsorption energy of lysozyme monomer at W/W interface (desorption energy) can be calculated as[11]

Supplementary Equation 1:

$$\Delta G_{mono} = \pi r^2 \gamma_{w/w}(1-|\cos\theta|)^2 \le \pi r^2 \gamma_{w/w} = 3.14\times(\frac{5nm}{2})^2 \times 10^{-5} N/m = 2\times 10^{-23} J$$

Where *r* is the hydrodynamic radius of lysozyme monomer, and θ is the contact angle. The maximum desorption energy is acquired when θ = 90°.

However, this adsorption energy is still much less than the kinetic energy driving the detachment of lysozyme monomer from the w/w interface, which is estimated as

Supplementary Equation 2:

$$\Delta E_k = kT = 1.38\times 10^{-23} J \bullet K^{-1} \times 300K = 4\times 10^{-21} J$$

This suggests the thermal kinetic energy dominates the motion of monomers, and the monomers can freely enter and leave the interface with negligible energy compensation. The situation is equally established for monomers at all contact angles (0°< θ<180°). According to

our theory, stabilizer of this kind can not form an effective barrier to prevent the direct contact of droplets or stabilize W/W emulsions.

Supplementary Note 2

A calculation of the minimum thickness of fibril coating suggesting the stabilized W/W emulsion droplets are coated by a monolayer of fibrils. When the concentrations of fibrils are kept at 0.025 wt%, 0.05 wt% and 0.075 wt%, the stabilized interfacial areas are approximately 0.02 m^2, 0.04 m^2 and 0.06 m^2, respectively (Fig. 2e in the main text). From the slope of the curve, 1 gram fibrils can stabilize an interfacial area of 0.02 m^2/0.025% = 80 m^2. Assuming the uniform distribution of fibrils at W/W interface, the weight of fibrils covering an interfacial area of 1 m^2 is 1/80 = 0.0125 g.

We suppose that the W/W emulsions are stabilized by fibril layers with a coverage ratio of C_f, then the weight of fibril layers coated on 1 m^2 of the W/W interface, W_{fib}, can also be expressed as,

Supplementary Equation 3: $W_{fib} = \rho_{fib} V = \rho_{fib} (1\ m^2 \times C_{fib} \times d) = 0.0125$ g,

where ρ_{fib} and V are the density and volume of the fibril layers; d is the height of fibril layers packed at the W/W interface. Density of fibril layers, ρ_{fib}, is estimated as 1.03 ± 0.02 g/cm^3. When a mixture solution of dextran, PEG, and fibrils is centrifuged at a high rotation rate (6500 rpm), a

fibril-rich phase is formed between the PEG-rich top phase and the dextran-rich bottom phase, suggesting the density of fibril-rich phase is between the dextran-rich (ρ_{dex} = 1.057 g/cm^3) and the PEG-rich phase (ρ_{PEG} = 1.01 g/cm^3). The coverage ratio of fibrils, C_{fib}, is approximately 60-70 %, which can be measured roughly from SEM images (Fig. 2f). Therefore, thickness of the fibril layers at the W/W interface, T_{fib} can be predicted as:

Supplementary Equation 4: $T_{fib} = W_{fib}/[\rho_{fib}\ (1\ \mathrm{m}^2 \times C_{fib}\ )] = 0.0125/(1.03\times10^6\times0.7) = 1.7\times10^{-8}$ m

The resultant thickness of fibril layer (17 nm) is approximately equal to the fibril width (15 nm), suggesting that stabilization of W/W emulsions require a monolayer of fibrils to cover the interface. This prediction is also confirmed from the SEM observation (Fig. 2d).

Supplementary Note 3

A model for calculating the adsorption energy of single fibril at the W/W interface

When a single piece of fibril leaves the W/W interface and enters the PEG-rich continuous phase, the adsorption/desorption energy can be estimated from the change in the total interfacial energy, as illustrated in the Supplementary Figure 3a. By accounting for the eliminated area of W/W interface ($S_{W/W}$) and the created PEG/fibril contact area ($S_{PEG/fib}$), the free energy change can be expressed as

Supplementary Equation 5: $\Delta G_{fibril} = \gamma_{w/w} S_{w/w} + S_{dex}(\gamma_{PEG/fib} - \gamma_{dex/fib})$

Ignoring the curvature of the W/W interface for simplicity, these areas are expressed as $S_{W/W} = 2RL\sin\alpha$ and $S_{dex} = 2\alpha RL$, where $2R$ (15 nm) is the cross-sectional diameter of fibrils, L (600 nm, Supplementary Figure 1) is the average length of fibrils and 2α is the angle subtended by the two three-phase contact lines. The fibril contact angle, θ, is the supplementary angle of α in the Supplementary Figure 3a, $\theta + \alpha = \pi$. Since the contact angle cannot be easily measured directly, due to the small sizes of fibrils, we deposited a film of lysozyme fibrils on a solid substrate and measured the wetting angle of a dextran-rich droplet at the PEG/fibrils interface. The measured wetting angle is $142° \pm 7°$ (see supplementary Figure 3b). This contact angle remains almost constant as the equilibrium concentrations of the two aqueous phases change. A static force equilibrium along the radial direction of the fibrils results in the following relationship,

Supplementary Equation 6: $\gamma_{PEG/fib} - \gamma_{dex/fib} + \gamma_{w/w}\cos(\pi - \theta) = 0$.

Combining Supplementary Equations 5 and 6, the adsorption energy can be expressed as,

Supplementary Equation 7: $\Delta G_{fibril} = 2R\gamma_{w/w}L(\sin\theta + \pi\cos\theta - \theta\cos\theta)$.

Supplementary Note 4

A physical model to calculate the energy barriers for coalescence of dextran-in-PEG emulsion and PEG-in-dextran emulsion.

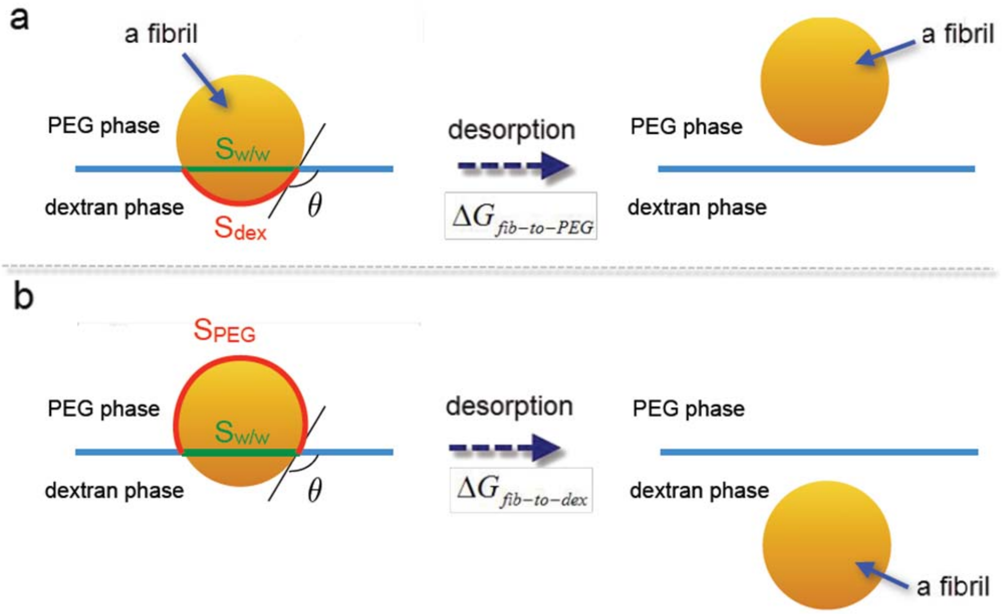

Supplementary Schematic 1: Desorption of fibrils from W/W interface when two adjacent droplet coalesce. (**a**) Coalescence of two PEG-in-dextran drops requires fibrils to detach from W/W interface and enter the PEG-rich droplet phase. The corresponding desorption energy is expressed as $\Delta G_{fib-to-PEG}$; (**b**) Coalescence of two dextran-in-PEG drops requires fibrils to detach from W/W interface and enter into the dextran-rich droplet phase. The desorption energy in this process is expressed as $\Delta G_{fib-to-dex}$.

Interfaces of two adjacent emulsion droplets must touch before they can merge into a single droplet. The role of fibrils is to form a physical barrier that separates the two interfaces. When two dextran-in-PEG emulsion drops approach with each other, the fibrils transiently desorb from W/W interface and enter either the PEG-rich continuous phase or the dextran-rich droplet phase. If fibrils transiently enter the PEG-rich continuous phase, they will separate the two dextran-rich droplets and prevent their direct contact. To coalesce, fibrils must enter the dextran-rich phase; however, this often requires consuming higher energy than entering to the PEG-rich phase, deduced from the calculation on the interfacial energy in the following paragraph.

When a single fibril transiently leave the W/W interface and enter the PEG-rich phase, the desorption energy can be estimated from the change in the total interfacial energy, as illustrated in Supplementary Schematic 1a. By accounting for the eliminated area of W/W interface ($S_{W/W}$) and the created PEG/fibril contact area ($S_{PEG/fib}$), the free energy change can be expressed as

Supplementary Equation 8: $\Delta G_{fib-to-PEG} = \gamma_{w/w} S_{w/w} + S_{dex}(\gamma_{peg/fib} - \gamma_{dex/fib})$

Assuming a flat interface, the contact area of a fibril to the dextran-rich phase is calculated as

Supplementary Equation 9: $S_{dex} = 2\alpha RL$.

Such that Supplementary Equation 8 becomes

Supplementary Equation 10: $\Delta G_{fib-to-PEG} = 2R\gamma_{w/w} L(\sin\theta + \pi\cos\theta - \theta\cos\theta) = 0.186\gamma_{w/w} RL$

Instead, when a single fibril transiently enters the dextran-rich emulsion phase, the associated desorption energy (see Supplementary Schematic 1b) can be estimated by

Supplementary Equation 11: $\Delta G_{fib-to-dex} = \gamma_{w/w} S_{w/w} + S_{peg}(\gamma_{dex/fib} - \gamma_{peg/fib})$

Assuming a flat interface, the contact area of a fibril to the PEG-rich phase is calculated as,

Supplementary Equation 12: $S_{PEG} = 2(\pi - \alpha)RL$.

Such that Supplementary Equation 11 becomes

Supplementary Equation 13: $\Delta G_{fib-to-dex} = \gamma_{w/w} DL[\sin\alpha + (\pi - \alpha)\cdot\cos(\pi - \theta)] = 5.14\gamma_{w/w} RL$

By comparing the desorption energy under the two conditions, the energetic cost is 25 times for getting a single fibril into the dextran-rich phase than to the PEG-rich phase. This explains why fibrils preferentially partition in the PEG-rich phase upon destabilization.

The calculation also denotes that the dextran-in-PEG emulsions must overcome a high energy barrier before coalescence can occur. In comparison, coalescence of PEG-in-dextran emulsions

occurs more easily because of the low energy barrier for fibrils to transiently enter the PEG-rich emulsion phase. Therefore, even with the same interfacial tension and calculated adsorption energy, fibril-coated dextran-in-PEG emulsion droplets are much more stable than the PEG-in-dextran emulsion droplets. However, when the interfacial tension declines to near zero, both of the two kinds of energy barrier becomes negligible compared to thermal energy. In this condition, fibrils almost distribute homogenously in the mixture.
